# *pncCCND1_B* Engages an Inhibitory Protein Network to Downregulate *CCND1* Expression upon DNA Damage

**DOI:** 10.3390/cancers14061537

**Published:** 2022-03-17

**Authors:** Ramona Palombo, Maria Paola Paronetto

**Affiliations:** 1 Laboratory of Molecular and Cellular Neurobiology, IRCCS Fondazione Santa Lucia, 00143 Rome, Italy; r.palombo@hsantalucia.it; 2 Department of Movement, Human and Health Sciences, University of Rome “Foro Italico”, Piazza Lauro de Bosis, 15, 00135 Rome, Italy

**Keywords:** Sam68, noncoding RNA, Ewing sarcoma, *CCND1*, DNA damage

## Abstract

**Simple Summary:**

Ewing sarcoma is a pediatric tumor characterized by chromosomal translocations, giving rise to the oncogene EWS-FLI1, which triggers the transcription of genes involved in neoplastic transformation including *CCND1*. In this work, we found that exposure to etoposide, a topoisomerase II inhibitor usually administered in combination with other drugs in the standard regimen for Ewing sarcoma treatment, induced cell death and reduced *CCND1* levels. Etoposide acts, at least in part, by enhancing the expression of the *pncCCND1_B*, a promoter-associated noncoding RNA transcribed from the promoter region of the *CCND1* gene. *PncCCND1_B* regulates in *cis* *CCND1* expression by forming a molecular complex with the RNA binding protein Sam68 and the DNA/RNA helicase DHX9. Upon exposure to etoposide, the increase in *pncCCND1_B* coupled with the decrease in DHX9 expression promote epigenetic changes and formation of DNA:RNA hybrids at the promoter region of *CCND1* gene, which downregulate its expression.

**Abstract:**

Promoter-associated noncoding RNAs (pancRNAs) represent a class of noncoding transcripts driven from the promoter region of protein-coding or non-coding genes that operate as cis-acting elements to regulate the expression of the host gene. PancRNAs act by altering the chromatin structure and recruiting transcription regulators. *PncCCND1_B* is driven by the promoter region of *CCND1* and regulates *CCND1* expression in Ewing sarcoma through recruitment of a multi-molecular complex composed of the RNA binding protein Sam68 and the DNA/RNA helicase DHX9. In this study, we investigated the regulation of *CCND1* expression in Ewing sarcoma cells upon exposure to chemotherapeutic drugs. Pan-inhibitor screening indicated that etoposide, a drug used for Ewing sarcoma treatment, promotes transcription of *pncCCND1_B* and repression of *CCND1* expression. RNA immunoprecipitation experiments showed increased binding of Sam68 to the *pncCCND1_B* after treatment, despite the significant reduction in DHX9 protein. This effect was associated with the formation of DNA:RNA duplexes at the *CCND1* promoter. Furthermore, Sam68 interacted with HDAC1 in etoposide treated cells, thus contributing to chromatin remodeling and epigenetic changes. Interestingly, inhibition of the ATM signaling pathway by KU 55,933 treatment was sufficient to inhibit etoposide-induced Sam68-HDAC1 interaction without rescuing DHX9 expression. In these conditions, the DNA:RNA hybrids persist, thus contributing to the local chromatin inactivation at the *CCND1* promoter region. Altogether, our results show an active role of Sam68 in DNA damage signaling and chromatin remodeling on the *CCND1* gene by fine-tuning transitions of epigenetic complexes on the *CCND1* promoter.

## 1. Introduction

Gene expression regulation is accomplished through a multistep process involving RNA polymerase II (RNAPII), transcription factors, and epigenetic modulators that affect promoter accessibility and RNAPII processivity [1]. RNA sequencing analysis revealed the presence of long and short noncoding RNAs (ncRNAs) transcribed from the promoter regions of most annotated genes. This class of ncRNAs display both stimulatory and inhibitory effects on the transcription of the host *loci* [2,3]. Promoter associated noncoding RNAs (pancRNAs) operate as *cis*-acting elements, displaying tissue specificity and contributing to many biological processes [3]. Gene expression changes occurring in several human genes have been linked to pancRNA differential expression [3]. Through their scaffolding function, pancRNAs affect the epigenetic signature of promoter sequences, thus impacting on gene expression [4]. The identification of specific noncoding RNAs (ncRNAs) arising from the promoter region of the *CCND1* gene unveiled a novel layer of regulation operated in *cis* by these noncoding transcripts, which are transcribed both in the sense and antisense direction from the same *locus* and display a specific and coordinated expression [4]. The most characterized of them is *pncRNA-D*, which is induced upon ionizing radiation (IR) and osmotic stress in Hela cells [4,5,6]. More recently, we have identified *pncCCND1_B* as a new epigenetic regulator in Ewing sarcoma malignancy [3,7]. They both negatively impact Cyclin D1 expression; nevertheless, the specificity of their molecular interactors determines some differences in the mechanism of action. Hence, the identification of RNA binding proteins (RBPs) that interact with them is critical to understanding how functional-converging pancRNAs employ alternative molecular strategies.

The Src-associated substrate in mitosis (Sam68) is a multifaceted RBP displaying a wide range of cellular functions and is involved in oncogenic transformation [8,9]. Sam68 participates in the transcription process via its interactions with transcription factors and epigenetic modifiers [7,10]. Moreover, we recently reported that Sam68 interacts with *pncCCND1_B* to repress *CCND1* transcription and Cyclin D1 expression in Ewing sarcoma cells [7]. This fine-tuned regulation is operated by DHX9 through alternative complexes formed with either EWS–FLI1 or *pncCCND1_B* and Sam68 [7].

To further dissect the regulatory mechanism underlying *CCND1* expression in Ewing sarcoma cells, we searched for molecules that induce transcription of *pncCCND1_B.* Herein, we report that etoposide treatment was able to upregulate *pncCCND1_B* expression and induce Sam68 re-localization to form a network hub on the *CCND1* promoter, contributing to *CCND1* downregulation. The molecular mechanism involves etoposide-induced DHX9 downregulation and the formation of DNA:RNA hybrids at the promoter region. In parallel, Sam68 interacts with HDAC1 and promotes the deacetylation of the nearby chromatin. Thus, Sam68 acts as a novel signaling molecule in the DNA damage response (DDR), coupling the compartmentalization in chromatin domains with the control of gene expression activity.

## 2. Materials and Methods

### 2.1. Cell Cultures and Drug Treatment

Ewing sarcoma cell lines TC-71 (RRID:CVCL_2213) and SK-N-MC (RRID:CVCL_0530) were purchased from DSMZ. Cell were grown in culture in Iscove’s modified Dulbecco’s medium (IMDM, GIBCO), supplemented with 10% fetal bovine serum, penicillin, and streptomycin (Gibco) in a humidified 37 °C incubator with a 5% CO_2_ atmosphere. Every two months, PCR analysis was performed to evaluate mycoplasma contamination. For the pan-inhibitor screening, Ewing sarcoma cells were treated for the indicated time with either DMSO or the indicated drug (see Table 1). Drugs were purchased from Selleckchem. Drug concentration: Afatinib (1 µM), Lapatinib (5 µM), Alisertib (5 µM), Barasertib (100 nM), Tozasertib (100 nM), PD0332991 (1 µM), Belnacasan (10 µM), Navitoclax (1 µM), KU-55933 (10 µM), JNK-IN-8 (1 µM), PF-562271 (1 µM), CPI-455 (10 µM), Etoposide (500 nM–10 mM), and BEZ-235 (1 µM). For UV light irradiation, cells were plated at 50–60% confluence 16 h before UV light irradiation (40 J/m^2^). Fresh medium was immediately added after the treatment and the cells were harvested after 6 h. Actinomycin D 10 µg/mL was used for the time course experiment.

### 2.2. Transfection Experiments

Over-expression experiments were performed by transfecting 1 μg/mL of pEGFP or pEGFP-DHX9 [25] using Lipofectamine 2000 (Invitrogen, Waltham, MA, USA). Twenty-four hours after transfections, cells were treated with either DMSO or etoposide and collected at the indicated time points for RNA or protein analyses, or for DRIP experiments. For the *pncCCND1_B* transfections, overexpression and silencing experiments were performed as previously described [7] using RNAimax reagent (Invitrogen) or Lipofectamine 2000 (Invitrogen), according to the manufacturer’s instructions.

### 2.3. SDS–PAGE and Western Blot Analyses

Protein extract preparation was performed as previously described [26]. Briefly, cells were washed twice with ice-cold phosphate buffered saline (PBS) and resuspended in RIPA lysis buffer in the presence of protease and phosphatase inhibitors (cocktail from Sigma-Aldrich). After 10 min on ice, soluble protein extracts were separated by centrifugation at 12,000 rpm for 10 min and diluted in Laemlli sample buffer. The obtained cell lysates were resolved on SDS–polyacrylamide gels (SDS-PAGE) and transferred on PVDF membrane Hybond TM-P (Amersham Bioscience, Amersham, UK). Membranes were saturated with 5% BSA at room temperature and incubated with the following primary antibodies at 4 °C overnight: mouse GAPDH (Santa Cruz Biotechnology, Dallas, TX, USA, SC-32233), mouse β-actin (Santa Cruz Biotechnology, Dallas, TX, USA, sc-47778), mouse GFP (B-2) (sc-9996), rabbit DXH9 (sc-66997), Cyclin D1 (sc-8396), mouse HDAC1 (sc-81598), mouse CBP/KAT3A/CREBBP (C-1) (sc-7300), rabbit Sam68 (Bethyl, Montgomery, TX, USA, A302-110A), rabbit gamma H2A.X (phospho S139) (Abcam, Cambridge, UK, ab11174), and rabbit PARP1 (Cell Signaling, Danvers, MA, USA, 9542). Secondary anti-mouse or anti-rabbit IgGs conjugated to horseradish peroxidase (Amersham) were incubated with the membranes for 1 h at room temperature at a 1:10,000 dilution. Immunostained bands were detected by a chemiluminescent method (Thermo Scientific, Waltham, MA, USA).

### 2.4. Reverse Transcription and Real-Time Quantitative PCR Analyses

RNA was isolated from Ewing sarcoma cells using the Trizol reagent (Invitrogen, Thermos Fisher Scientific, Waltham, MA, USA). After DNase digestion, 1 μg of total RNA was reverse transcribed by Reverse Transcriptase M-MLV (Promega, Madison, WI, USA) following the manufacturer’s instructions. PCR reactions were run in triplicate on a QuantStudio1 Real Time qPCR instrument (Thermos Fisher Scientific, Waltham, MA, USA). For all experiments, no-RT controls were performed. PCR amplification was carried out with 1 μL of the 1:10 diluted reverse transcription sample with 10 μL of 2× Luna Universal qPCR Master Mix (New England Biolabs -NEB) and 4 pmol of specific gene primer pairs in a 20-μL total volume in 96-well microtiter plates.

Primers: (*pncCCND1_B* Fw: 5′-TGAGATTCTTGGCCGTCTGT-3′; *pncCCND1_B* Rev 5′-CCATATCCAAGCCGGCAGA-3′; *CCND1*-Fw 5′-GTGCAAGGCCTGAACCTG-3′; *CCND1*-Rev 5′-CGGGTCACACTGATCACTC-3′; *GAPDH* Fw: 5′-TGGTCACCAGGGCTGCTT-3′; *GAPDH* Rev 5′-CATGTAGTTGAGGTCAATGAAGG-3′).

### 2.5. Immunoprecipitation

Immunoprecipitation experiments were performed as previously described [7]. Briefly, TC-71 cells were homogenized in lysis buffer (100 mM NaCl, 10 mM MgCl_2_, 50 mM Hepes, 2 mM EDTA, pH 8, 10% glycerol, 1 mM DTT, protease inhibitor cocktail, 1mM dithiothreitol, 0.5 mM Na-ortovanadate, 1%, 10 mM B-glycerophosphate, and 10 mM sodium fluoride) supplemented with 1% Triton X-100. Protein extracts were isolated by centrifugation at 10,000× *g* for 10 min. For immunoprecitipation, 1 mg of protein extracts were incubated with Dynabeads conjugated with Protein A (Invitrogen, Thermos Fisher Scientific, Waltham, MA, USA) and 1 μg of mouse HDAC1 (sc-81598) or purified rabbit IgGs (Sigma Aldrich, St. Louis, MO, USA), overnight at 4 °C under constant rotation. Dynabeads were washed three times with lysis buffer and eluted in Laemlli buffer for western blot analysis.

### 2.6. Chromatin Immunoprecipitation

ChIP experiments were performed as previously described [25]. A total of 2 × 10^8^ TC-71 cells were cross-linked with 1% (*w*/*v*) formaldehyde for 15 min at room temperature, followed by formaldehyde inactivation with the addition of 125 mM glycine. Isolated nuclei were extracted (extraction buffer: 1% SDS, 10 mM EDTA, and 50 mM Tris-HCl (pH 8.0), 1 mM dithiothreitol, 10 mM β-glycerophosphate, 0.5 mM Na_3_VO_4_, and protease inhibitor cocktail (Sigma-Aldrich, St. Louis, MO, USA)) and sonicated with Bioruptor (Dyagenode, Seraing, Belgium) 2 × 6 min (30 s sonication and 30 s pause). Samples were centrifuged at 10,000× *g* for 10 min at 4 °C to pre-clear the chromatin. Supernatants containing DNA fragments (50 μg/sample) with an average size of 200–300 bp were pre-cleared for 2 h overnight on Protein A/agarose/salmon sperm DNA (Millipore, Burlington, MA, USA) and then immunoprecipitated overnight using 1 μg of anti-acetylated H3 (Merck Millipore, Burlington, MA, USA), H3 (Novus Biologicals, NB500-171), or FLI1 (Abcam, Cambridge, UK, ab15289). Isolated DNA was extracted using PCI (phenol:chloroform:isoamyl alcohol, Ambion, AM9730), precipitated and analyzed by qPCR, with the following primer pairs: *CCND1* promoter Fw 5′-AGGTGTGTTTCTCCCGGTTA—3′ and *CCND1* promoter Rev 5′-CTGCCTTCCTACCTTGACCA—3′, *pncCCND1_B* promoter Fw 5′-CCCAGGACCCGGAATATTAGTAA—3′ and *pncCCND1_B* promoter Rev 5′-AGGGTGCTCACAGCAAGATG—3′.

### 2.7. In Vivo Crosslinking and Immunoprecipitation (CLIP)

The CLIP assay was performed as previously described [27,28]. Briefly, TC-71 cells were cross-linked by irradiation (150 mJ/cm^2^) in a Stratalinker 2400 at 254 nm, on ice. Cells were harvested and incubated for 10 min on ice in lysis buffer (50 mM Tris, pH 7.4, 100 mM NaCl, 1% Igepal CA-630 (Sigma-Aldrich, St. Louis, MO, USA, I8896), 0.1% SDS, 0.5% sodium deoxycholate, 0.5 mM Na_3_VO_4_, 1-mM DTT, protease inhibitor cocktail (Sigma-Aldrich, St. Louis, MO, USA), and RNase inhibitor (Promega) for the extraction of protein–RNA complexes. Samples were sonicated at medium intensity for 3′ (6 × 30″) and incubated with 10 μL of 1/1000 RNase I (Ambion, Thermos Fisher Scientific) dilution and 2 μL Turbo DNase (Ambion, Thermos Fisher Scientific) for 3 min at 37 °C under constant shaking, and then centrifuged at 15,000× *g* for 10 min at 4 °C. One mg of extract was immunoprecipitated using rabbit anti-Sam68 antibody or purified IgGs (negative control) in the presence of Protein A magnetic Dynabeads (Life Technologies, Carlsbad, CA, USA). Immunoprecipitates were incubated overnight at 4 °C under constant rotation. After stringent washes with high salt buffer (50 mM Tris-HCl, pH 7.4, 1 M NaCl, 1 mM EDTA, 1% Igepal CA-630 (Sigma Aldrich, St. Louis, MO, USA), 0.1% SDS, 0.5% sodium deoxycholate), beads were equilibrated with PK buffer (100 mM Tris-HCl, pH 7.4, 50 mM NaCl, 10 mM EDTA) and treated with 50 µg Proteinase K for 20′ at 37 °C. Next, 7 M urea was added for a further 20 min at 37 °C. The RNA was extracted with phenol/CHCl_3_ (Ambion, Thermos Fisher Scientific) and precipitated overnight at −20 °C with 0.5 μL glycoblue (Ambion, Thermos Fisher Scientific), 3 M sodium-acetate pH 5.5, and 100% ethanol. *pncCCND1_B* binding was evaluated by RT-qPCR after retro-transcription of immunoprecipitated RNA, with the following primer pair: *pncCCND1_B* Fw: 5′-TGAGATTCTTGGCCGTCTGT -3′; *pncCCND1_B* Rev 5′-CCATATCCAAGCCGGCAGA-3′).

### 2.8. Immunofluorescence Analysis

TC-71 cells were fixed in 4% paraformaldheyde (PFA) and washed with PBS, then stained for immunofluorescence analysis. Briefly, cells were permeabilized with 0.1% Triton X-100 for 10 min and incubated for 2 h in 5% BSA. Cells were then washed with PBS and incubated for 2 h at room temperature (RT) with anti-Sam68 (rabbit, Bethyl—A302-110A, 1:1000), and anti-HDAC1 (mouse, Santa Cruz—sc-81598, 1:1000) antibodies, followed by 1 h of incubation with FITC-conjugated anti-rabbit IgGs (Alexa-Fluor, Thermo Fisher Scientific), or TRITC-conjugated anti-mouse IgGs (Alexa-Fluor, Thermo Fisher Scientific). DAPI was used for DNA staining. Cells were washed with PBS and mounted with Mowiol (Calbiochem). Immunostained cells were analyzed by confocal microscopy. All solutions were prepared in nuclease-free water. Immunofluorescence image acquisition was performed using a Zeiss confocal microscope. Images were analyzed using ZEN2 Blue edition software (Zeiss). Colocalization analysis was performed on a “pixel by pixel” basis using ImageJ JACoP plugin. The colocalization coefficients were measured for each channel and calculated by summing the pixels in the colocalized region and then dividing by the sum of pixels in each channel. Each pixel had a value of 1 [29]. Scatterplot images were obtained by Zeiss analysis software (ZEN2 Blue edition software), in which every pixel of a selected region of interest (ROI) is plotted in the scatter diagram based on its intensity level from each channel [29].

### 2.9. DNA: RNA Hybrid Immunoprecipitation (DRIP) and Chromatin-Associated RNA Immunoprecipitation (CARIP)

DRIP and CARIP experiments were performed as previously described [30,31]. Ewing sarcoma cells were washed twice at room temperature with PBS. For the cross-link, formaldehyde (Sigma-Aldrich, St. Louis, MO, USA) was added to a final concentration of 1%, incubated for 15 min at RT, and quenched by the addition of chilled glycine (Sigma-Aldrich, St. Louis, MO, USA), as for the ChIP experiment. Nuclei were isolated with hypotonic buffer (5 mM PIPES (pH 8.0), 85 mM KCl, NP40 0.5%, 1 mM dithiothreitol, 10 mM β-glycerophosphate, 0.5 mM Na_3_VO_4_, and protease inhibitor cocktail (Sigma-Aldrich, St. Louis, MO, USA)) and lysed in two different buffers for the DRIP and CARIP experiments. For the DRIP experiment, a buffer containing 1% SDS, 10 mM EDTA, 50 mM Tris-HCl (pH 8.0), 1 mM dithiothreitol, 10 mM β-glycerophosphate, 0.5 mM Na_3_VO_4_, protease inhibitor cocktail (Sigma-Aldrich, St. Louis, MO, USA), and RNase inhibitor (Promega, Madison, WI, USA) was used. For the CARIP experiments, nuclear pellets were re-suspended in 1X TE (Tris-EDTA), 0.1% SDS, RNase inhibitor (Promega, Madison, WI, USA), 1 mM dithiothreitol, 10 mM β-glycerophosphate, 0.5 mM Na_3_VO_4_, and protease inhibitor cocktail (Sigma-Aldrich, St. Louis, MO, USA). The samples were sonicated with a Bioruptor (Dyagenode, Seraing, Belgium) for 2 × 4 min (30 s sonication and 30 s pause) to obtain a partial fragmentation of the chromatin, and then pre-cleared by centrifugation at 10,000× *g* at 4 °C for 10 min. For the negative control, supernatants containing sonicated chromatin were digested for 30 min at 37° C with RNAse H (5 U/sample) (Thermos Fisher Scientific, EN0202). Agarose/salmon sperm DNA Protein A and G were mixed and washed with sonication buffers. One μg of S9.6 antibody (Sigma-Aldrich, St. Louis, MO, USA, MABE1095) or mouse IgGs were added to beads and incubated in constant rotation for 1 h at 4 °C. Antibody-pre-absorbed beads were incubated overnight with pre-cleared samples under constant rotation at 4 °C. De-crosslinking procedure was performed by incubating washed beads with 1% SDS, 0.1 M NaHCO_3_, at 28 °C for 30 min, and then treated with 50 µg Proteinase K and incubated overnight at 65 °C, under constant 1100 rpm shaking. DNA (DRIP experiment) and RNA (CARIP experiment) extraction was performed either in PCI (phenol:chloroform:isoamyl alcohol, Ambion, AM9730) or phenol:chloroform (Ambion, Thermo Fisher Scientific, AM9720), respectively, following the manufacturer’s instructions. RNA was reverse transcribed by using Reverse Transcriptase M-MLV (Promega). DNA and cDNA were analyzed by Real Time-qPCR. For the DRIP experiments, the following primers were used: *pncCCND1_B* F 5′-TGAGATTCTTTGGCCGTCTGT-3′ and *pncCCND1_B* R 5′-CCATATCCAAGCCGGCAGA-3′, and for the CARIP experiments: *pncCCND1_B* Fw: 5′-TGAGATTCTTGGCCGTCTGT-3′ and *pncCCND1_B* Rev 5′-CCATATCCAAGCCGGCAGA-3′.

### 2.10. Statistical Analysis

The Student t-test and analysis of variance (ANOVA) were used to determine significant differences. To evaluate statistical differences among groups, a post hoc analysis with Bonferroni corrections was performed. Data analysis was performed using Prism GraphPad. Significant differences are denoted by *p*-values < 0.05. Pearson’s and Mander’s correlation coefficients were evaluated using the ImageJ JACoP plugin.

## 3. Results

### 3.1. Pan-Inhibitor Screening Unveils Etoposide as a Modulator of pncCCND1_B Expression

**Figure 1:**
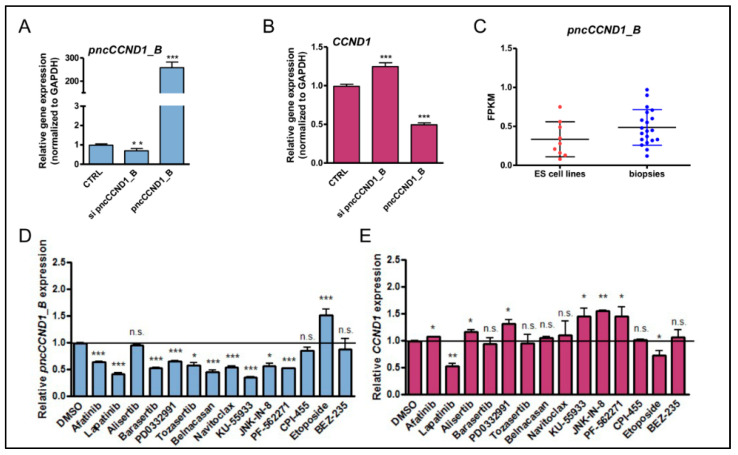
Pan-inhibitor screening unveils etoposide as a modulator of *pncCCND1_B* expression. The expression of *pncCCND1_B* (**A**) and *CCND1* mRNA (**B**) was monitored in TC-71 Ewing sarcoma cells after transfection with either the control or *sipncCCND1_B* oligonucleotides or in vitro transcribed *pncCCND1_B*. Histograms represent three independent experiments (±S.D.). Statistical analysis was performed by the Student’s *t*-test versus CTRL. (**C**.) Expression levels of *pncCCND1_B* were evaluated in a dataset derived from an RNA-sequencing experiment of a total of 20 Ewing sarcoma patients and nine Ewing sarcoma cell lines (dbGaP accession phs000804.v1.p1) [7,36]. The expression of *pncCCND1_B* (**D**) and *CCND1* mRNA (**E**) was monitored in TC-71 Ewing sarcoma cells after different 16 h drug treatments. Histograms represent three independent experiments (±S.D.). Statistical analysis was performed by the Student *t*-test versus DMSO. *p*-value (* < 0.05, ** < 0.01, *** < 0.005, n.s. > 0.05).

We previously identified the promoter associated noncoding RNA *pncCCND1_B* as a regulator of *CCND1* expression [7]. Notably, *pncCCND1_B* overexpression or siRNA-mediated downregulation were sufficient to modulate CCND1 expression in Ewing sarcoma cells (Figure 1A,B). Importantly, analysis of *pncCCND1_B* expression in multiple Ewing sarcoma cell lines and patients showed similar levels (Figure 1C), suggesting that the former are suitable tools for mechanistic investigation of the regulation of *pncCCND1_B* expression.

Next, given the key role of Cyclin D1 in cell proliferation and cancer [32], we asked whether pharmacological manipulation of pncCCND1_B expression could be exploited to modulate Cyclin D1 expression. To this end, we carried out a pan-inhibitor screening to identify drugs able to affect *CCND1* expression by modulating *pncCCND1_B* transcription. At first, we determined the *pncCCND1_B* half-life by blocking de novo transcription with actinomycin D, and then monitoring the decay of *pncCCND1_B* over time (Supplementary Figure S1A). RT-qPCR analysis revealed that the *pncCCND1_B* transcript was almost halved in 8 h, while 16 h was required to reach the minimum transcript level. This time point was chosen for the screening. TC-71 Ewing sarcoma cells were treated for 16 h with several chemotherapeutic agents widely used in cancer therapy, and are listed in Table 1. RT-qPCR analysis showed a significant decrease in *pncCCND1_B* after treatment with most of the chemotherapeutic agents, with a significant 1.5-fold upregulation of *CCND1* expression after treatment with KU 55933, JNK-IN-8, and PF-562271 (Figure 1D,E). Conversely, only etoposide treatment was able to induce a significant 1.5-fold increase in *pncCCND1_B* expression, together with a corresponding decrease in *CCND1* mRNA (Figure 1D,E). A similar regulation was also observed in SK-N-MC Ewing sarcoma cells (Supplementary Figure S1B,C), which express lower levels of Cyclin D1 than TC-71 cells (Supplementary Figure S1D), suggesting a general effect in Ewing sarcoma.

**Figure 2:**
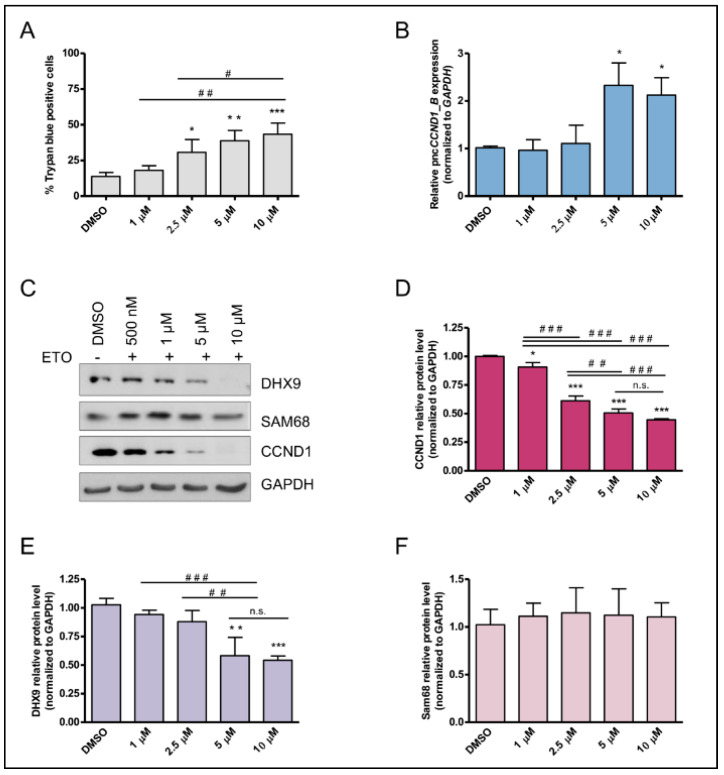
Etoposide treatment inversely affects *pncCCND1_B* and CCND1 in a dose dependent fashion. (**A**) Percentage of blue positive cells by Trypan blue staining at 16 h after treatment with different concentrations of Etoposide. (**B**) RT-qPCR analysis of *pncCCND1_B* expression at different etoposide concentrations, as in A. (**C**) Western blot analysis upon treatment with either DMSO (vehicle) or increasing concentration of etoposide (ETO). After 16 h of treatment, DHX9, Sam68, and CCND1 protein levels were analyzed in protein total lysates (15 µg). GAPDH was used as the loading control. Histograms represent CCND1 (**D**), DHX9 (**E**), and Sam68 (**F**) protein levels normalized to the GAPDH signal and relative to DMSO. Statistical analysis was performed by ANOVA with Bonferroni correction. *p*-value (*, ^#^ < 0.05, **, ^##^ < 0.01, ***, ^###^ < 0.005, n.s. > 0.05).

Etoposide is a component of multiagent chemotherapy critical for the management of Ewing sarcoma patients [33]. We found that etoposide induced cell death in a dose dependent fashion in both TC-71 (Figure 2A) and SK-N-MC cells (Supplementary Figure S2A), as revealed by Trypan blue staining. This effect correlated with the increase in *pncCCND1_B* (Figure 2B), and the decrease in Cyclin D1 protein (Figure 2B,C and Supplementary Figure S2B,C). Since the *pncCCND1_B* was shown to form a multimolecular complex composed by Sam68 and DHX9 [7], we evaluated the expression of these RBPs upon etoposide treatment. Notably, etoposide induced a strong decrease in DHX9 expression (Figure 2C,E and Supplementary Figure S2B,D). Under these conditions, the expression of Sam68 was not affected in both the TC-71 (Figure 2C,F) and SK-N-MC (Supplementary Figure S2B,E) cells. Remarkably, a similar effect on *pncCCND1_B* induction and DHX9 expression was also observed upon irradiation with low doses (40 J/m^2^) of UV light (Supplementary Figure S3A–C). Since both treatments cause DNA-damage induced inclusion of the poison exon 6A in the *DHX9* transcript [25,34], which leads to nonsense-mediated mRNA decay (NMD) [35], these results suggest a causal link between DHX9 reduction and *pncCCND1_B* induction.

Collectively, these experiments show that etoposide treatment induces upregulation of *pncCCND1_B* and downregulation of *CCND1* in Ewing sarcoma cells. In this condition, the DHX9-Sam68 complex cannot form due to the marked decrease in DHX9 expression.

### 3.2. Etoposide Induces DNA:RNA Hybrids at the CCND1 Promoter

**Figure 3:**
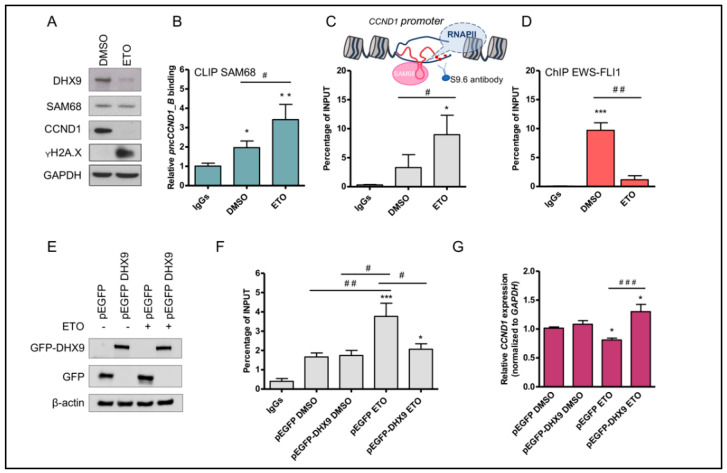
Etoposide induces the formation of DNA:RNA hybrids at *CCND1* promoter. (**A**) Western blot analyses were performed on 15 μg of total extracts of TC-71 cells treated for 16 h with either DMSO (vehicle) or 5 µM etoposide (ETO). DHX9, Sam68, CCND1, and γH2A. X protein levels were analyzed. GAPDH was used as loading control. (**B**) Histograms represent Sam68 binding to *pncCCND1_B* from cross-linked and immunoprecipitation experiments. Values are expressed as input percentage and normalized to IgGs signal. (**C**) qPCR analysis of S9.6 immunoprecipitated chromatin from DNA:RNA heteroduplex in the promoter region of the *CCND1* gene. As represented in the illustration, the S9.6 antibody recognizes DNA:RNA hybrids. (**D**) EWS-FLI1 recruitment on the *CCND1* promoter was evaluated by ChIP experiments. Histograms represent input percentage from three independent experiments. (**E**) Western blot analysis to monitor recombinant expression of GFP or GFPDHX9 in TC-71 cells, treated with either DMSO or etoposide 5 μM. Ten μg of total extract was loaded in each lane. (**F**) qPCR analysis of S9.6 immunoprecipitated chromatin from DNA:RNA heteroduplexes in the promoter region of the *CCND1* gene. Histograms represent input percentage. (**G**) RT-qPCR analysis of *CCND1* expression in TC-71 cells transfected with either GFP or GFP-DHX9, and treated with DMSO or etoposide. Histograms represent three independent experiments (±S.D.). Statistical analysis was performed by ANOVA with Bonferroni post hoc test. *p*-value (*, ^#^ < 0.05, **, ^##^ < 0.01, ***, ^###^ < 0.005).

Etoposide induces a signal transduction cascade that affects chromatin architecture and gene expression programs [37]. Emerging evidence suggests that RBPs play critical functions in DNA damage signaling [38,39,40] including Sam68 [41,42]. Thus, we asked whether etoposide-induced DNA damage signaling could affect Sam68 binding to the *pncCCND1_B*. Cross-linked and immunoprecipitation (CLIP) experiments documented increased binding of Sam68 to the *pncCCND1_B* after etoposide treatment (Figure 3B). Since DHX9 has been shown to play a key role in regulating R-loop accumulation [43,44], we also asked whether the etoposide-induced downregulation of DHX9 could affect the formation of DNA:RNA hybrids on the *CCND1* promoter. DNA:RNA hybrid immunoprecipitation (DRIP) experiments with the S9.6 antibody, which specifically recognizes DNA:RNA hybrids, confirmed the increase in DNA:RNA hybrids on the *CCND1* promoter upon etoposide treatment (Figure 3C). Accumulation of DNA:RNA hybrids correlated with reduced recruitment of EWS-FLI1 on the *CCND1* promoter, as revealed by the Chromatin immunoprecipitation (ChIP) experiments (Figure 3D). Similar effects were also observed upon irradiation with low doses of UV light, whereas BEZ-235, which was not able to modulate *pncCCND1_B* expression in our screening, did not induce DNA:RNA hybrids (Supplementary Figure S4). To test whether increased formation of DNA:RNA hybrids at the CCND1 promoter was due to reduced DHX9 function, we performed rescue experiments. Overexpression of recombinant GFP-DHX9, but not GFP alone, was sufficient to solve DNA:RNA hybrids and recover CCND1 expression in etoposide treated cells (Figure 3E,F).

These experiments suggest that DHX9 downregulation causes an increase of DNA:RNA hybrids on the CCND1 promoter upon etoposide treatment, which leads to the inhibition of EWS-FLI1 recruitment.

### 3.3. Etoposide Effect on SAM68-pncCCND1_B Complex Is Abolished by KU 55933 Treatment

**Figure 4:**
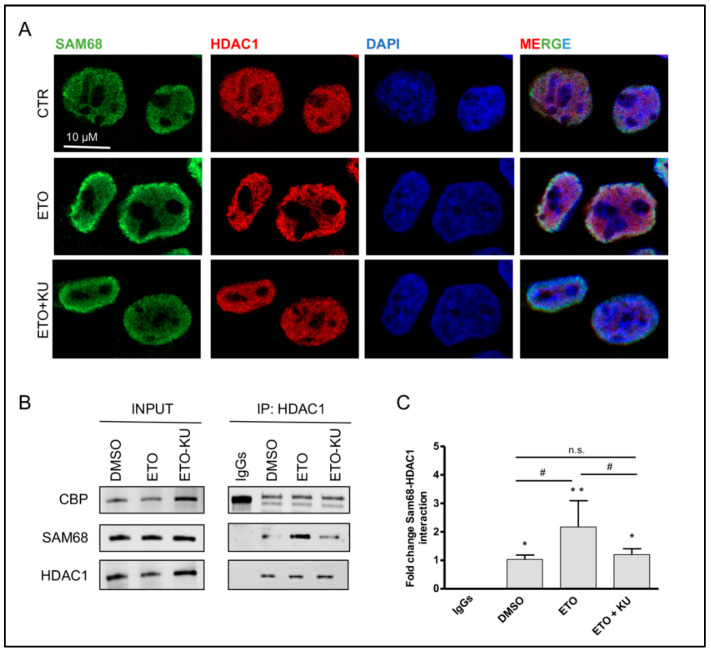
Sam68 and HDAC1 participate in the DNA damage response. (**A**) Immunofluorescence analysis was performed on TC-71 cells treated with either DMSO, 5 µM etoposide (ETO), or etoposide with pre-treatment of 10 µM of KU55933 (ETO + KU). (**B**) Western blot analysis of co-immunoprecipitation experiments performed from nuclear extracts of TC-71 cells treated with DMSO (vehicle), 5 µM etoposide (ETO), or etoposide with pre-treatment of 10 µM of KU 55,933 (ETO + KU). HDAC1 protein was immunoprecipitated with a specific antibody; CBP protein was used as the positive control. Sam68 was detected in the immunoprecipitated proteins. (**C**) Histograms represent the densitometric analysis of Sam68-HDAC1 interaction in each condition relative to DMSO, from three independent experiments. Statistical analysis was performed by ANOVA with the Bonferroni post hoc test. *p*-value (*, ^#^ < 0.05, ** < 0.01, n.s. > 0.05).

Sam68 is a signaling RBP, whose post-translational modifications deeply affect its localization and RNA binding properties [8,45]. Notably, imposed genotoxic stress causes Sam68 accumulation in nuclear foci [41]. Moreover, Sam68 can be recruited to DNA lesions, where it interacts with PARP1 [42] and regulates DNA damage-initiated PAR production [42]. Since the DNA damage response is signaled by the ATM signal transduction pathway, we asked whether inhibition of this pathway plays a role in Sam68 localization upon etoposide treatment. Immunofluorescence analysis showed Sam68 relocalization to the nuclear lamina at 16 h after treatment (Figure 4A). This effect was mitigated by the ATM inhibitor KU 55933. Notably, histone deacetylase activity has been involved in gene silencing at the nuclear lamina [46]. In particular, HDAC1 was proposed as a key factor required for histone H3 and H4 deacetylation and chromatin silencing at nuclear lamina-associated domains [46]. We found that HDAC1 was partially relocalized to the chromatin domains nearby the lamina upon etoposide treatment. The correlation analysis revealed that nuclear areas of more intense Sam68 fluorescence signal were associated with a parallel increase in HDAC1 staining, with a 0.62 Pearson’s correlation coefficient and a 0.75 Mander’s coefficient of co-staining between Sam68 and HDAC1 upon etoposide treatment (Supplementary Figure S5A–C). Co-immunoprecipitation experiments confirmed the association between Sam68 and HDAC1 in etoposide-treated cells, which was reduced by the inhibition of ATM activity (Figure 4B,C, Figure S7).

**Figure 5:**
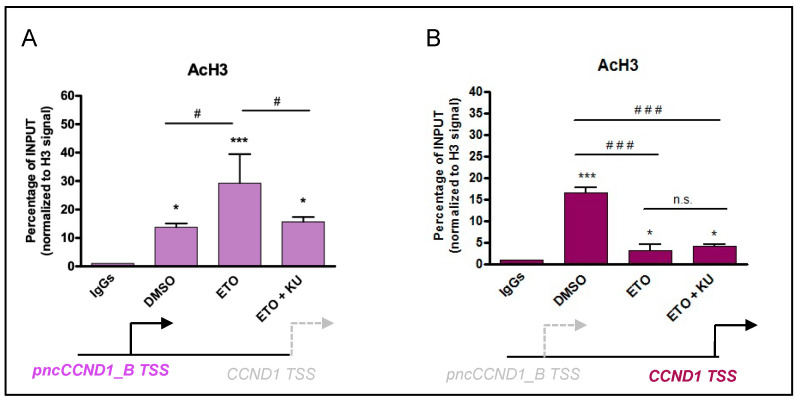
Histone acetylation of *CCND1* promoter upon etoposide treatment. Histone acetylation was evaluated by ChIP analysis upon DMSO (vehicle), 5 µM etoposide (ETO), or etoposide with pre-treatment of 10 µM of KU 55933 (ETO + KU). The acetylated status of the promoter was measured by qPCR analysis at the *pncCCND1_B* promoter (upstream region, (**A**)) and at a downstream region (**B**), close to transcription start site (TSS) of the *CCND1* gene. *p*-value was evaluated by ANOVA with Bonferroni correction (*, ^#^ < 0.05, ***, ^###^ < 0.005, n.s. > 0.05).

Histone deacetylation mediated by HDAC1 recruitment could repress transcription. To test this hypothesis, we performed ChIP experiments to monitor histone H3 acetylation. Under basal conditions, we detected high chromatin acetylation in the promoter region of *CCND1* (Figure 5, right panel) whereas acetylation of regions upstream of the *pncCCND1_B* transcription start site was very low (Figure 5, left panel). In contrast, etoposide treatment caused a strong reduction in H3-acetylation of the *CCND1* promoter whereas the upstream region corresponding to the *pncCCND1_B* promoter displayed increased acetylation. These results parallel the increase in *pncCCND1_B* expression and the decrease in *CCND1* transcript (Figure 5). Interestingly, inhibition of ATM signaling via KU 55933 was sufficient to inhibit etoposide-induced acetylation of the *pncCCND1_B* promoter (Figure 5A), but it did not rescue the acetylation of the *CCND1* promoter (Figure 5B).

**Figure 6:**
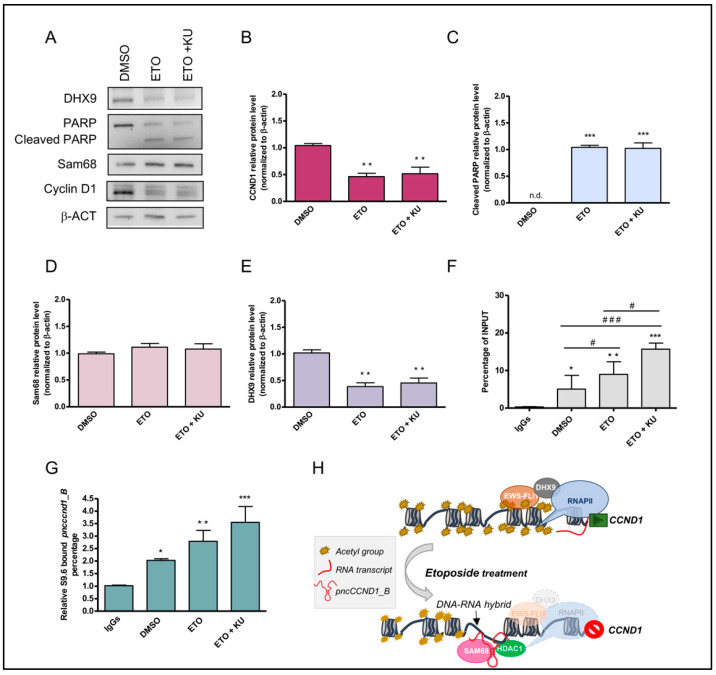
DNA:RNA heteroduplex formation, caused by DHX9 repression, impairs transcription at the *CCND1* promoter. (**A**) Representative western blot upon treatment with DMSO (vehicle), 5 µM etoposide (ETO), and combination of etoposide and KU 55933 (10 µM) (ETO + KU). Histograms represent DHX9 (**B**), cleaved PARP1 (**C**) (n.d. not detectable), Sam68 (**D**), and *CCND1* (**E**) protein levels normalized to β-actin, relative to DMSO. (**F**) qPCR analysis of the promoter region of *CCND1* gene on S9.6 immunoprecipitated chromatin (DRIP experiment). (**G**) RT-qPCR analysis of *pncCCND1_B* in DNA:RNA immunoprecipitated heteroduplexes (CARIP experiment). Statistical analysis was performed by ANOVA with Bonferroni correction ANOVA. (**B**–**G**) *p*-value (*, ^#^ < 0.05, ** < 0.01, ***, ^###^ < 0.005). (**H**) Schematic representation of the *CCND1* promoter region. Acetylation of histone proteins is required for the transcription of *CCND1* gene by EWS-FLI1. This oncoprotein interacts with the DNA/RNA helicase DHX9, facilitating RNAPII recruitment and *CCND1* transcription. Etoposide treatment enhances the transcription of the *pncCCND1_B*, whereas it inhibits the expression of *CCND1* through the formation of an inhibitory complex due to the formation of RNA:DNA hybrid structures.

Western blot analysis confirmed Cyclin D1 downregulation after inhibition of ATM signaling (Figure 6A,B). Under these conditions, the maintenance of PARP1 cleavage indicates the permanence of DNA breaks in the absence of ATM activity (Figure 6A,C). Western blot analysis also showed that Sam68 expression was not modulated by etoposide or KU 55933 treatment (Figure 6A,D) whereas a reduction in DHX9 protein was maintained (Figure 6A,E), suggesting that the RNA:DNA hybrids at the *CCND1* promoter were not resolved. Indeed, DRIP experiments showed that the DNA:RNA hybrids persisted in the presence of KU 55933 treatment (Figure 6F). Moreover, analysis of RNAs associated with the hybrids at the CCND1 promoter confirmed the presence of *pncCCND1_B* (Figure 6G). The formation of DNA:RNA hybrids was also confirmed in the SK-N-MC cells, highlighting a general regulatory mechanism in Ewing sarcoma (Supplementary Figure S6).

Collectively, these experiments document a novel role for the *pncCCND1_B* upon etoposide treatment in determining chromatin deacetylation on the *CCND1* promoter through the recruitment of Sam68 and HDAC1.

## 4. Discussion

In this study, we identified and validated etoposide as a regulator of *pncCCND1_B* expression and activity on the *CCND1* promoter in Ewing sarcoma cells. Ewing sarcoma is the second most common primary bone cancer in pediatric patients [47]. It is a highly aggressive mesenchymal tumor with the tendency to develop metastasis [47]. The standard therapy for patients relies on multidisciplinary regimens, which include surgical removal, radiotherapy, and administration of a multidrug chemotherapy, displaying high toxicity and deleterious long-term effects. Etoposide is included in the standard regimen adopted for Ewing sarcoma treatment. Hence, deciphering the mechanistic insights of its effects could be instrumental in fine-tuning the therapy.

We describe here that etoposide induces upregulation of *pncCCND1_B* in a dose dependent fashion, paralleling the recruitment of the RBP Sam68 on *pncCCND1_B* RNA and promoting *CCND1* downregulation. The described mechanism relies on Sam68 interaction with HDAC1 and deacetylation of the *CCND1* promoter. Sam68 (Src-associated substrate during mitosis of 68 kDa) is a versatile RBP that plays a role in several cellular processes including RNA stability, splicing, nuclear export, HIV-1 replication, adipogenesis, neuronal activity, and others [8,9,48,49,50,51]. Sam68 was also identified as a PAR-binding protein [52], playing a crucial function in PARP1 activation upon DNA damage [42]. The interaction between Sam68 and PARP1 is critical for DNA dependent-PARP1 activation both in vivo and in vitro [42]. It was also shown that DNA damage is poorly repaired in Sam68-deficient cells and animals [42]. Moreover, depletion of Sam68 sensitized prostate cancer cells to etoposide-induced cell death [53]. These studies indicated that Sam68 plays an important role in the maintenance of genome integrity.

In this work, we identified HDAC1 as a novel partner of Sam68 in the DNA damage response. We found that Sam68 interacts with HDAC1 and they partially relocalize to the nuclear lamina after etoposide treatment. Notably, the nuclear lamina makes extensive contacts with chromatin not engaged in nucleoplasmic RNAPII transcription [54], thus reflecting the intrinsic inactive property of these domains [55]. Mammalian gene *loci* display nonrandom positioning within the cell nucleus, and specific chromosome territories appear to play a role in their functional regulation [56]. Silencing of gene clusters can involve interactions with the nuclear lamina [57,58], whereas loss of these interactions can lead to transcriptional activation, indicating that proximity to the lamina may define gene repression [58]. In line with these findings, our data indicate local deacetylation of the *CCND1* promoter after etoposide treatment, paralleling Sam68 and HDAC1 relocalization at the nuclear lamina. This local deacetylation is also maintained after the inhibition of ATM by KU 55933. However, etoposide-induced acetylation of the region upstream of the *pncCCND1_B* RNA transcription start site was abolished upon ATM inhibition and disruption of the Sam68-HDAC1 complex, suggesting the existence of a complex regulatory network at the *CCND1* promoter region, which modulates differential acetylation of neighboring chromatin domains in response to etoposide-induced DNA lesions.

We previously showed that Sam68 forms a multimolecular complex with the *pncCCND1_B* and DHX9 [7]. Overexpression of *pncCCND1_B* in Ewing sarcoma cells led to significant downregulation of the *CCND1* transcript, whereas its silencing, although reducing the expression only of 40%, led to a slight but significant increase in *CCND1* expression. However, upon etoposide treatment or UV light irradiation, DHX9 expression was strongly repressed due to the inclusion of a poison exon in *DHX9* mRNA [34], which targets *DHX9* transcript to NMD [25,34]. Conversely, BEZ-235, which did not affect *pncCCND1_B* and *CCND1* expression in our screening, did not cause DNA:RNA hybrid formation on the *CCND1* promoter. However, we cannot exclude that other features triggered by etoposide could also be observed upon inhibition of the PI3K/AKT/mTOR pathway by BEZ-235.

DHX9 is involved in various aspects of transcription and RNA metabolism [59]. DHX9 impacts transcription either by direct binding to RNA [60], binding to promoters [61], DNA/RNA unwinding activity [62,63], or by the coordination of protein networks [7,64,65]. In Ewing sarcoma, DHX9 promotes EWS-FLI1 transcriptional activity and contributes to oncogenic transformation [63,64]. Remarkably, DHX9 also interacts with DNA:RNA hybrids and resolves them [65], thus preventing R-loop accumulation [44]. Therefore, DHX9 may be recruited to the promoters of transcribed genes through different mechanisms, where it can then suppress the accumulation of R-loops, thus maintaining genomic integrity in response to genotoxic stress. In line with this notion, we found that downregulation of DHX9 induced by etoposide or low doses UV light irradiation led to R-loop formation on the *CCND1* promoter, which also persisted upon inhibition of ATM signaling. It would be interesting to evaluate whether other DNA damage inducing drugs display similar effects as etoposide and UV light irradiation.

Indeed, R-loop accumulation is a common feature of cancer cells, and multiple members of the DEAD/H helicase family including DHX9 are strongly enriched and frequently deregulated in a wide range of cancers [44] including Ewing sarcoma [34]. Thus, DHX9 might also be required to support the higher transcriptional and metabolic activity of cancer cells by preventing R-loop accumulation. In this context, the effect of etoposide on DHX9 expression could be instrumental to hamper cancer cell proliferation and metastasis.

## 5. Conclusions

In conclusion, our work provides novel mechanistic insights on the regulation of *CCND1* expression by *pncCCND1_B* and RBP Sam68 upon etoposide treatment, highlighting the relevance of DHX9 activity in preventing R-loop formation. Remarkably, pharmacogenomics analysis suggested that the expression of RNA:DNA hybrid binding proteins in cancer is associated with survival and resistance to therapeutic treatments [66]. Thus, deciphering the mechanistic insights underlying R-loop formation in cancer cells could help to define novel therapeutic strategies to sensitize cancer cells to treatments. In this direction, our results open the path to epigenetic-based therapies tailoring R-loop levels in the *CCND1* gene.

## Figures and Tables

**Table 1:** Small molecule inhibitors used in the pan inhibitor screening.

Compound Name	Concentration	Biological Activity	References
AFATINIB	1 μM	Irreversible inhibitor of EGFR, HER2 and HER4	[11,12]
LAPATINIB	5 μM	Inhibitor of EGFR and HER2 tyrosine kinase domain	[13]
ALISERTIB	100 nM	Selective inhibitor of the Aurora-A kinase	[14]
BARASERTIB	20 nM	Inhibitor of Aurora-B and Aurora-A kinases	[15]
PD0332991	100 nM	CDK4/6 inhibitor	[16]
TOZASERTIB	100 nM	Pan-Aurora inhibitor	[17]
BELNACASAN	10 μM	Selective caspase-1 inhibitor	[18]
NAVITOCLAX	1 μM	High-affinity inhibitor of BCL-2	[19]
KU 55933	10 μM	ATM inhibitor	[20]
JNK-IN-8	1 μM	Irreversible JNK inhibitor for JNK1, JNK2 and JNK3	[21]
PF-562271	1 μM	ATP-competitive, reversible inhibitor of FAK	[22]
CPI-455	10 μM	TNF alpha inhibitor	[23]
ETOPOSIDE	5 μM	Topoisomerase II inhibitor	[24,25]
BEZ-235	100 nM	Dual PI3K/mTOR inhibition	[26]

## Data Availability

The data presented in this study are available on request from the corresponding author.

## Supplementary Materials

The following are available online at https://www.mdpi.com/article/10.3390/cancers14061537/s1, Figure S1: Etoposide treatment affects *pncCCND1_B* expression in SK-N-MC cells; Figure S2: Etoposide treatment affects Cyclin D1 expression in SK-N-MC cells; Figure S3: UV light irradiation affects *CCND1* expression in Ewing sarcoma cells; Figure S4: UV light irradiation induces DNA:RNA hybrids in Ewing sarcoma cells; Figure S5: Sam68 colocalizes with HDAC1 upon etoposide treatment; Figure S6: KU-55933 pre-treatment does not resolve etoposide-induced DNA:RNA heteroduplex formation in SK-N-MC cells; Figure S7: Original Western blots.
