# Supplementary material for: pncCCND1_B Engages an Inhibitory Protein Network to Downregulate CCND1 Expression upon DNA Damage
Source: Cancers (Basel). 2022 Mar 17;14(6):1537. doi: 10.3390/cancers14061537 (PMC8946712; doi:10.3390/cancers14061537)
Supplement: Supplementary file 1 [file cancers-14-01537-s001.zip › cancers-1616006-supplementary.pdf]

Supplementary Materials: *pncCCND1_B* Engages an Inhibitory Protein Network to Downregulate *CCND1* Expression upon DNA Damage

Ramona Palombo and Maria Paola Paronetto

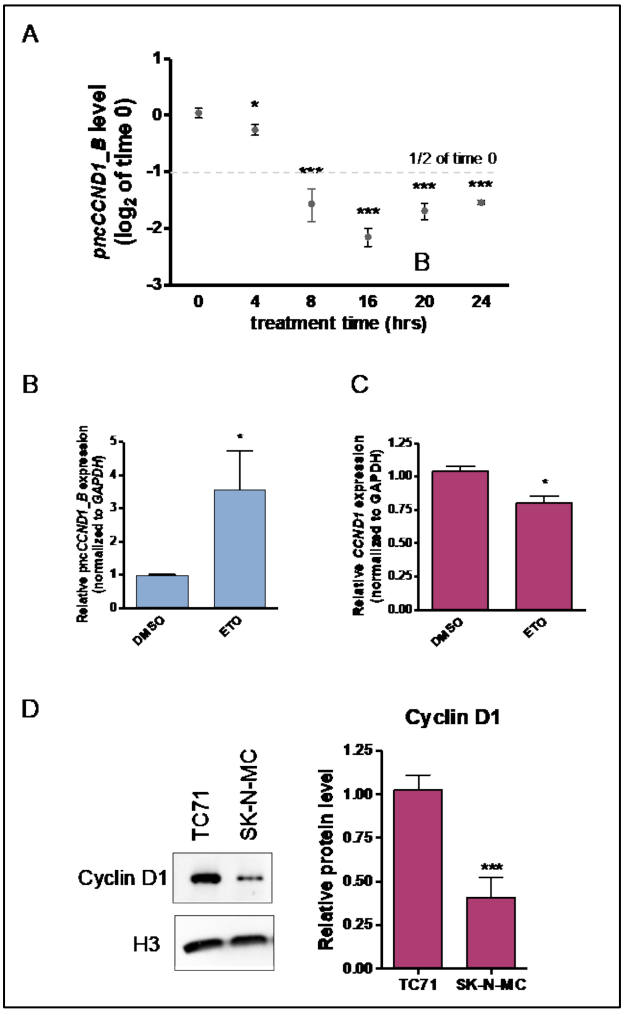

Figure S1. Etoposide treatment affects *CCND1* expression in SK-N-MC cells. (A) RT-qPCR analysis to monitor *pncCCND1_B* expression, at different time points after actinomycin D block of transcription. Significance was determined by Student's *t*-test for each point. *p* value: *$p < 0.05$; ***$p < 0.001$. Expression of *pncCCND1_B* (B) and *CCND1* mRNA (C) was monitored in SK-N-MC Ewing sarcoma cells after 16 h of 2.5 mM Etoposide treatment. Histograms represent three independent experiments (S.D.) *p* value (* $p < 0.05$). (D) On the left, representative western blot to compare Cyclin D1 expression in TC-71 and SK-N-MC cells. 10µg of total extracts were loaded in each lane. H3 antibody was used as loading control. Histograms represent relative protein level normalized to H3 content. Student's *t*-test *p* value: ***$p < 0.001$.

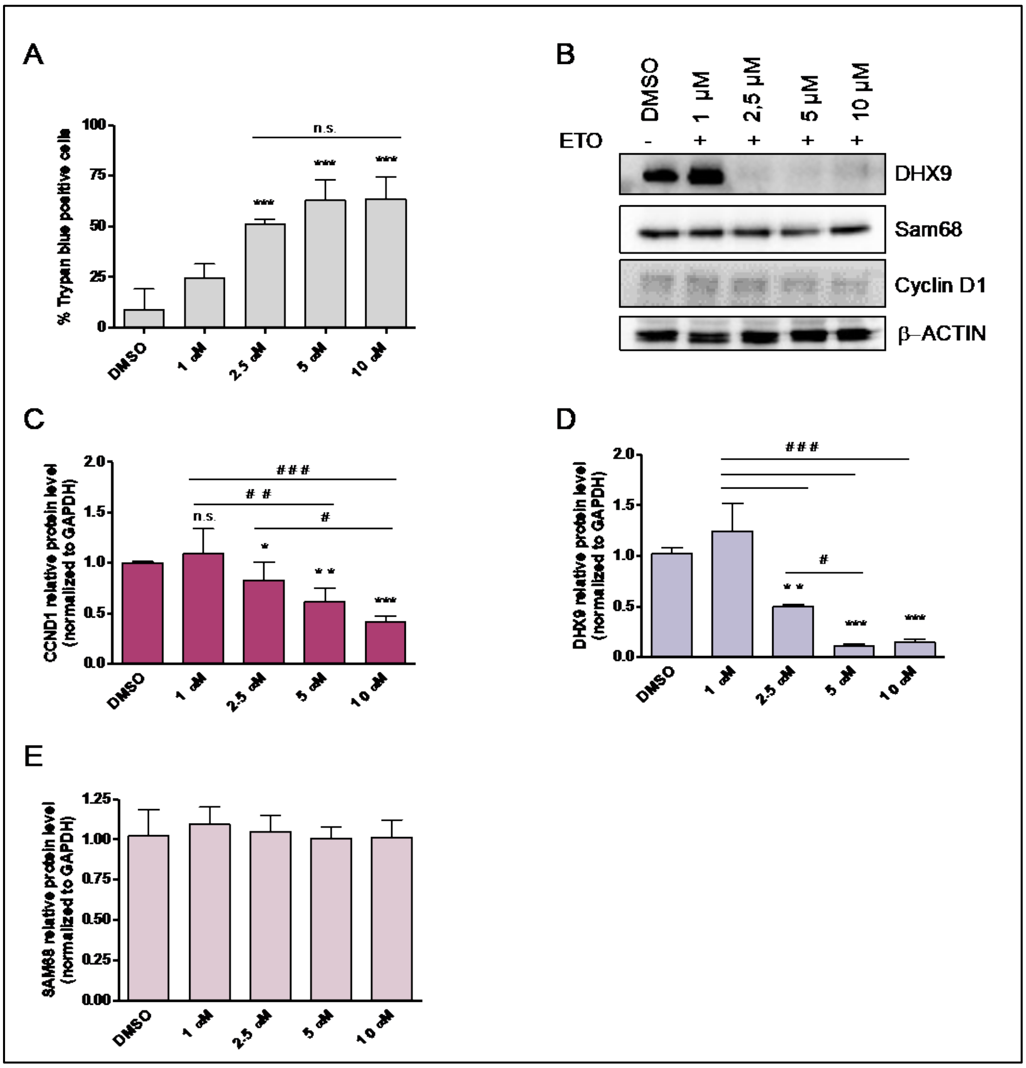

Figure S2. Etoposide treatment affects Cyclin D1 expression in SK-N-MC cells. (A) Histograms represent the percentage of dead cells after increasing concentration of Etoposide. Statistical analysis was performed by Student's *t*-test: ****p* < 0.001 for CTR vs etoposide treatment. (B) Western blot upon treatment with either DMSO (vehicle) or increasing concentration of Etoposide (ETO). After 16 hours of treatment, DHX9, SAM68 and Cyclin D1 protein levels were analyzed in total cell lysates (15 μg). β-actin was used as loading control. Histograms represent Cyclin D1 (C), DHX9 (D), and Sam68 (E) protein levels normalized to β-actin signal and relative to DMSO. *p*-value (*, $^{\#}p < 0.05$, **, $^{\#\#}p < 0.01$, ***, $^{\#\#\#}p < 0.001$).

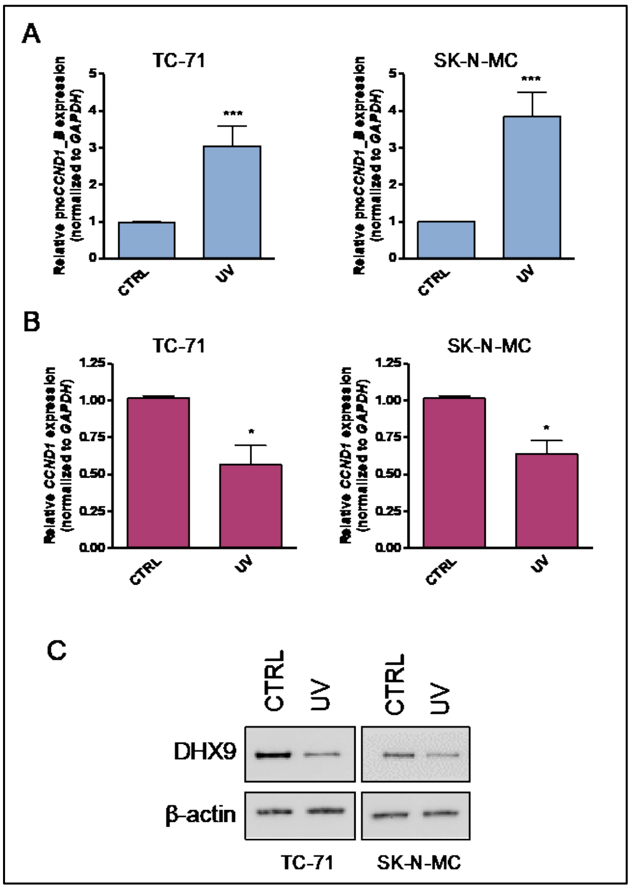

Figure S3. UV light irradiation affects CCND1 expression in Ewing sarcoma cells. RT-qPCR analysis to monitor *pncCCND1_B* (A) and *CCND1* (B) transcripts in TC-71 and SK-N-MC Ewing sarcoma cells upon 40 J/m² UV light irradiation. Cells were harvested 6 hours after irradiation for RNA extraction. Histograms represent three independent experiments (mean ± S.D.). Student's *t*-test *p* value: **p* < 0.05, ****p* < 0.001. (C) Western Blot analysis to monitor DHX9 expression upon 40 J/m² UV light irradiation. Cells were harvested 6 hours after irradiation for protein extraction. 10 µg of protein extracts were loaded in each lane. β-actin was used as loading control.

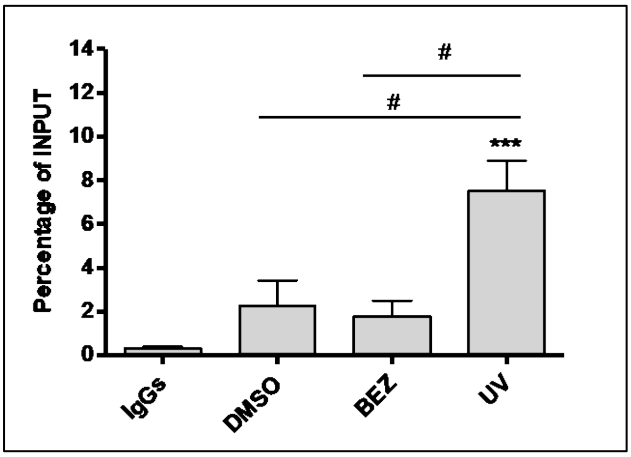

Figure S4. UV light irradiation induces DNA:RNA hybrids in Ewing sarcoma cells . Histograms represent qPCR analysis of S9.6 immunoprecipitated DNA/RNA heteroduplex in the promoter region of *CCND1* gene upon treatment with either DMSO or BEZ-235 (BEZ), or after UV light irradiation (40J/m²). Histograms represent three independent experiments (mean ± S.D.). Bonferroni corrected ANOVA *p* value: ****p* < 0.001 vs IgGs; # *p* < 0.05 among groups.

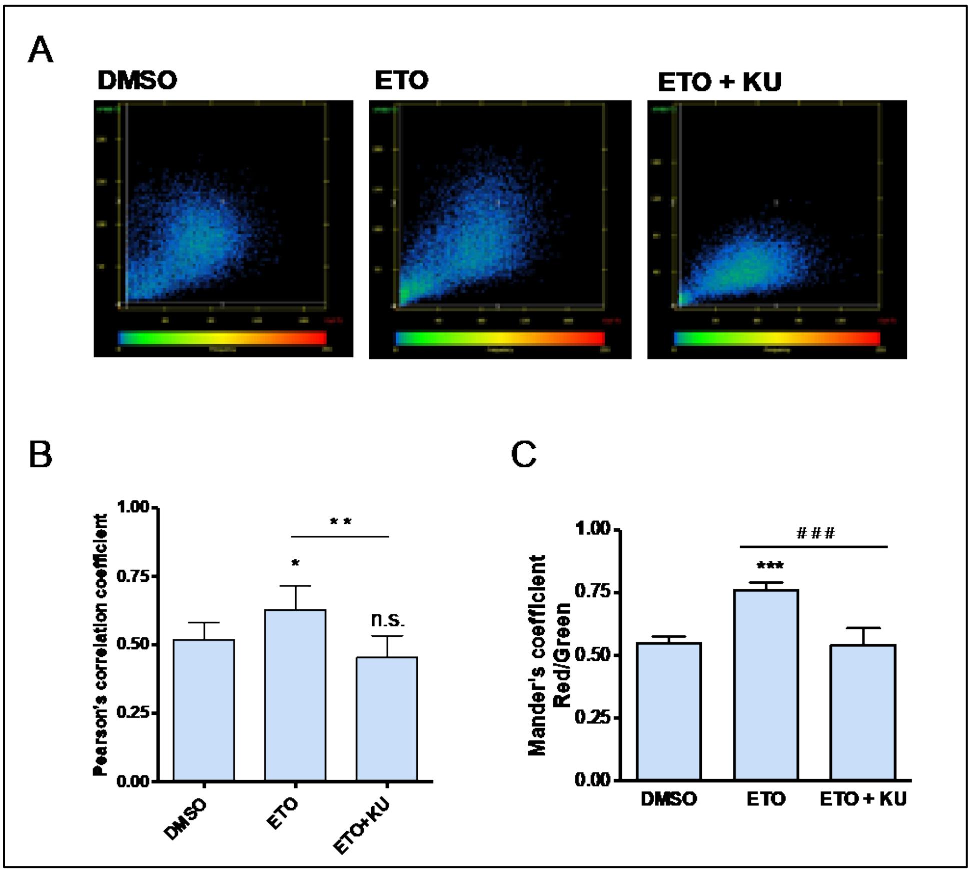

Figure S5. Sam68 colocalizes with HDAC1 upon etoposide treatment. (A) Representative scatterplots of immunofluorescence images, for specific regions of interest (ROIs). Pixels intensity for green fluorescence (Sam68) are assigned to y-axis, and red fluorescence (HDAC1) to x-axis. (B) Pearson's correlation coefficient was measured for 10 ROIs in different images, for each condition. *p*-value (* $p < 0.05$, ** $p < 0.01$, n.s.>0.05). (C) Mander's correlation coefficient was measured for 10 ROIs in different images, for each condition. *p*-value (*** $p < 0.001$ versus DMSO: ### $p < 0.001$ among treatments).

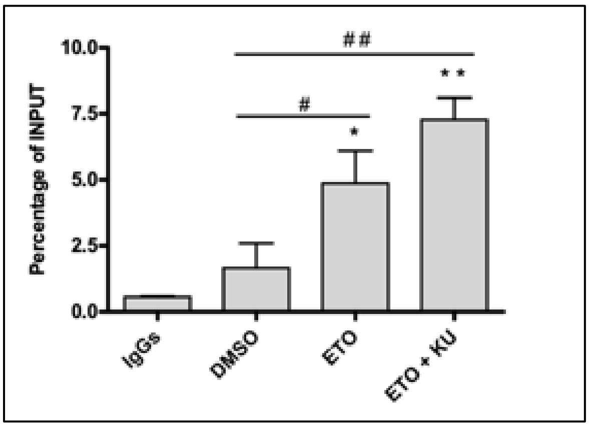

Figure S6. KU-55933 pre-treatment does not resolve etoposide-induced DNA:RNA heteroduplexes formation in SK-N-MC cells. Histograms represent qPCR analysis of S9.6 immunoprecipitated DNA:RNA heteroduplex in the promoter region of *CCND1* gene. Analysis was performed upon DMSO, 2.5 µM Etoposide, or 2.5 µM Etoposide after 1 hour of pre-treatment with 10 µM KU-55933. Histograms represent three independent experiments (mean ± S.D.). Bonferroni corrected ANOVA *p* value: *$p < 0.05$, **$p < 0.01$, vs IgGs; $^{\#}p < 0.05$, $^{\#\#}p < 0.01$ among groups.

Fig. 2C

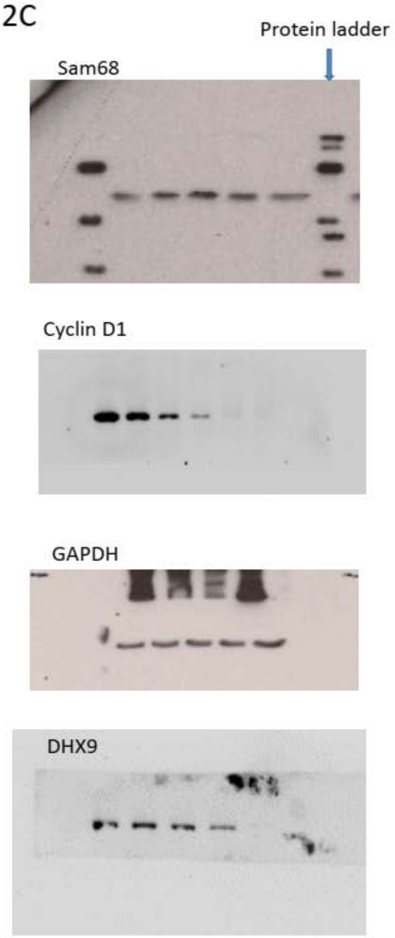

Fig. 3A

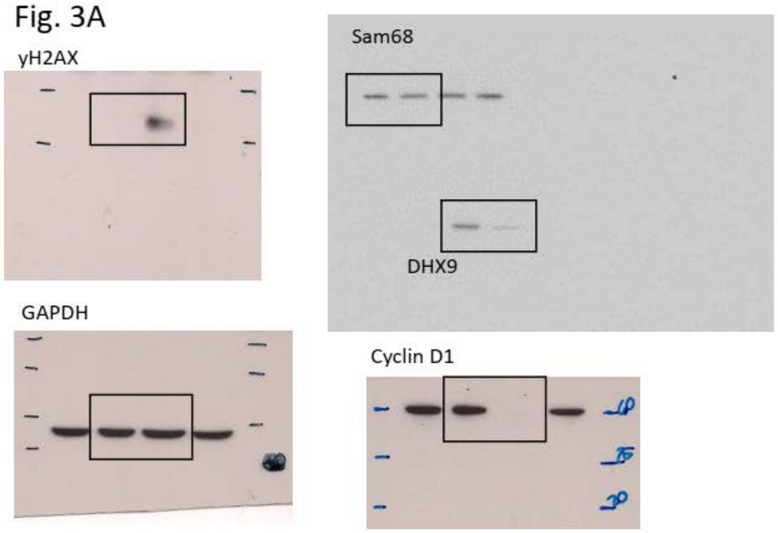

Fig. 3E

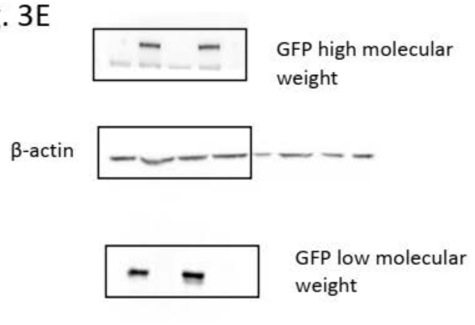

Fig. 4B

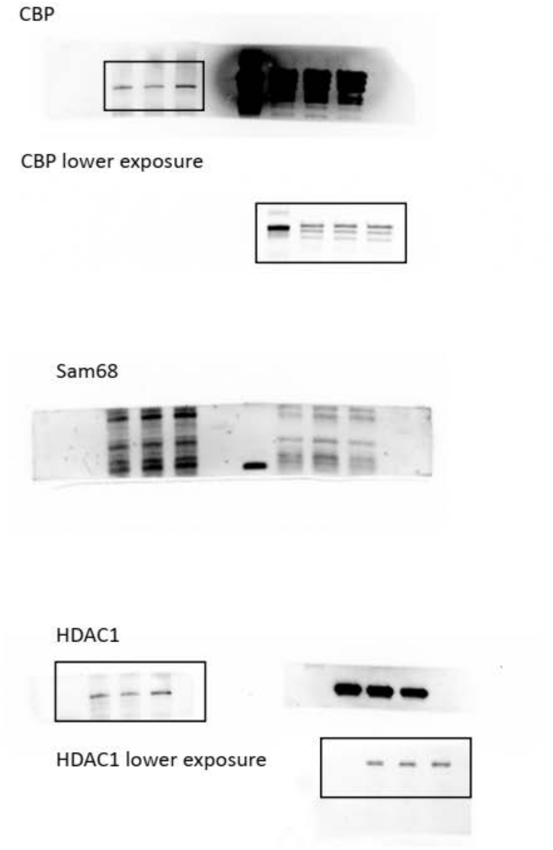
CBP
CBP lower exposure
Sam68
HDAC1
HDAC1 lower exposure

Fig. 6A

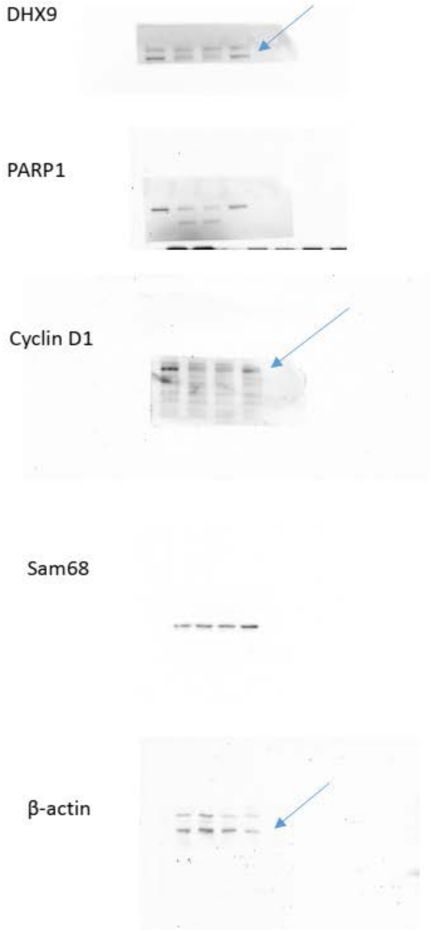
DHX9
PARP1
Cyclin D1
Sam68
β-actin

Suppl Fig. 2B

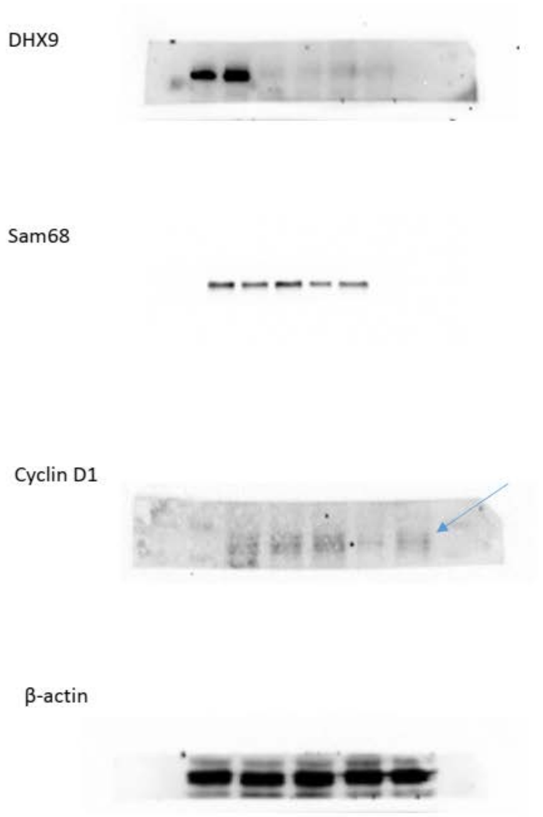

Suppl Fig. 3C

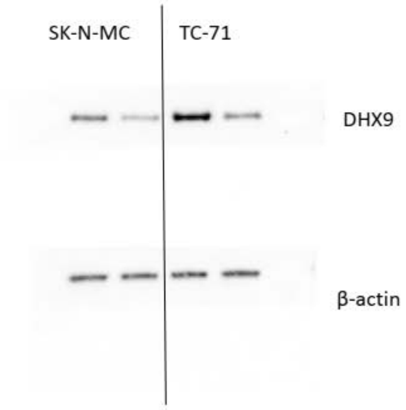

Figure S7: Original Western blots.
